# Exploring the role of oxidative stress and mitochondrial dysfunction in β-damascone-induced aneuploidy

**DOI:** 10.1186/s41021-024-00319-3

**Published:** 2024-11-25

**Authors:** Tsuneo Hashizume, Satoru Munakata, Tomohiro Takahashi, Taku Watanabe

**Affiliations:** Scientific Product Assessment Center, Japan Tobacco Inc., 6-2, Umegaoka, Aoba-Ku, Yokohama, Kanagawa 227-8512 Japan

**Keywords:** Aneugenicity, β-damascone, Micronucleus formation, Mitochondrial dysfunction, Oxidative stress

## Abstract

**Background:**

The rose ketone β-damascone (β-Dam) elicits positive results in the in vitro micronucleus (MN) assay using human lymphocytes, but shows negative outcomes in the Ames test and combined in vivo MN and comet assays. This has led to the interpretation that the in vitro MN result is a misleading positive result. Oxidative stress has been suggested as an indirect mode of action (MoA) for in vitro MN formation, with the α, β-unsaturated carbonyl moiety of the β-Dam chemical structure expected to cause misleading positive results through this MoA. In this study, we investigated the role of oxidative stress in β-Dam-induced in vitro MN formation by co-treatment with the antioxidant *N*-acetyl-l-cysteine (NAC), thereby highlighting a possible link between mitochondrial dysfunction and aneugenicity.

**Results:**

β-Dam induced MN formation in both CHL/IU and BEAS-2B cells, with the response completely inhibited by co-treatment with NAC. Moreover, β-Dam induced oxidative stress-related reporter activity in the ToxTracker assay and increased reactive oxygen species levels, while decreasing glutathione levels, in BEAS-2B cells in the high-content analysis. All of these effects were suppressed by NAC co-treatment. These findings indicate that β-Dam elicits oxidative stress, which causes DNA damage and ultimately leads to MN induction. However, no significant DNA damage-related reporter activities were observed in the ToxTracker assay, nor was there an increased number of γH2AX foci in the high-content analysis. These data suggest that MN formation is not a DNA-reactive MoA. Considering recent reports of aneuploidy resulting from chromosome segregation defects caused by mitochondrial dysfunction, we investigated if β-Dam could cause such dysfunction. We observed that the mitochondrial membrane potential was dose-dependently impaired in BEAS-2B cells exposed to β-Dam.

**Conclusions:**

These findings suggest that the oxidative stress induced by β-Dam exposure may be explained through an aneugenic MoA via mitochondrial dysfunction, thereby contributing to MN formation in mammalian cells.

**Supplementary Information:**

The online version contains supplementary material available at 10.1186/s41021-024-00319-3.

## Introduction

Assessing the genotoxic potential of chemicals, such as pharmaceuticals, agrochemicals, pesticides, and food additives, is a critical step in ensuring their relative safety under intended conditions of use [1]. A comprehensive assessment of genotoxicity includes a battery of tests to evaluate mutagenicity, clastogenicity, and aneugenicity [2, 3]. Some chemicals that cause chromosomal damage in vitro may not elicit the same response in vivo, resulting in potentially misleading positive results. Traditionally, in vivo follow-up testing has been the preferred approach to confirm the genotoxic potential of chemicals that show positive in vitro outcomes [4, 5]. However, in recent times, in vivo follow-up testing has been considered a final option because of concerns around animal welfare, regulatory restrictions, and cost and time limitations [6]. This potentially results in promising chemicals being omitted from the innovation pipeline.

**Fig. 1:**
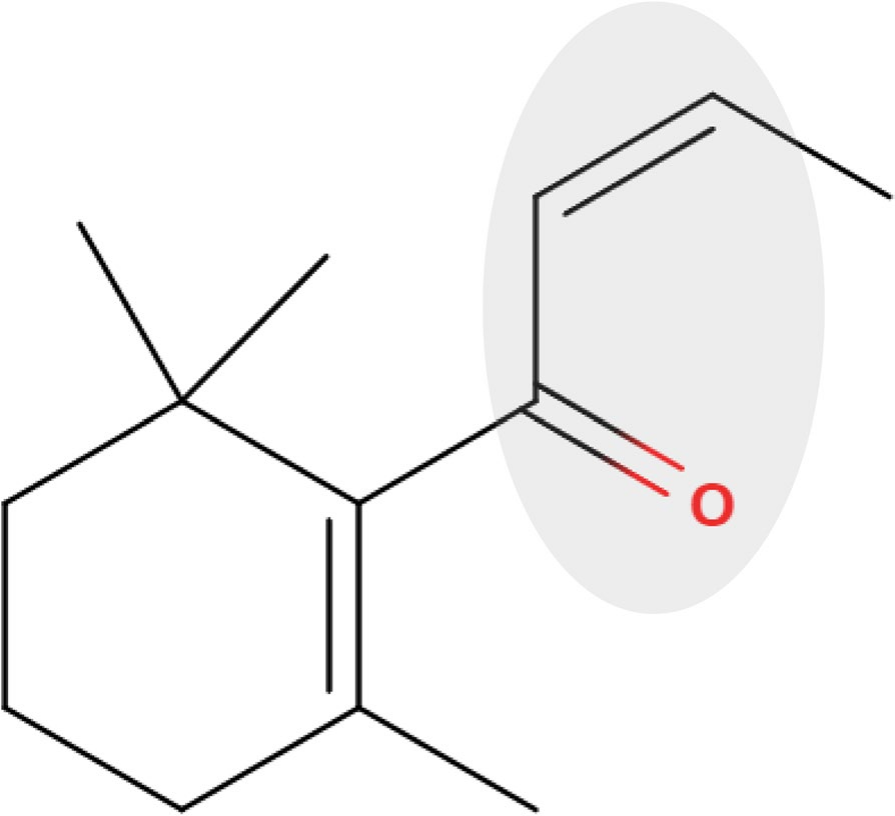
Chemical structure of β-damascone (β-Dam). The α, β-unsaturated carbonyl moiety, highlighted in light grey, is recognized as the key chemical structure of β-Dam that is responsible for inducing oxidative stress

To address the potential for misleading positive results, it is essential to thoroughly investigate the genotoxic mode of action (MoA) in vitro and assess the necessity for additional in vivo follow-up studies based on the 3Rs principle (Replacement, Reduction, and Refinement), while considering human relevance. Indirect genotoxic MoAs, such as inhibition of topoisomerase II, inhibition of DNA synthesis, chemical binding to aurora kinases, tubulin binding, and increased reactive oxygen species (ROS) levels, have the potential to induce chromosomal damage [7–9]. Chemicals that contain an α, β-unsaturated carbonyl moiety are anticipated to form adducts and deplete cysteine-containing reducing agents, such as glutathione (GSH). This can lead to an imbalance between oxidants and antioxidants in the cell, called oxidative stress, and eventually result in DNA modifications as a genotoxic MoA [10–12]. One such chemical is the rose ketone β-damascone (β-Dam) [13], illustrated in Fig. 1, which is widely used as a flavoring agent in foods [14].

Although β-Dam elicited positive results in the micronucleus (MN) assay when tested on human lymphocytes, negative results were obtained with the Ames test and combined in vivo MN and comet assays, as reported by the European Food Safety Authority (EFSA) [14]. To elucidate whether oxidative stress underlies the capacity of β-Dam to induce MN formation, several mechanistic approaches, including the ToxTracker assay [15], ROS generation measurement [16, 17], and reduced GSH depletion assessment [18], could offer valuable insights. Because *N*-acetyl-l-cysteine (NAC) serves as a precursor of GSH biosynthesis and functions as an antioxidant itself [19, 20], co-treatment or supplementation with NAC may prove beneficial for mitigating GSH depletion and ROS generation during oxidative stress.

In our previous study [18], we demonstrated that NAC pretreatment attenuated the genotoxicity and cytotoxicity induced by allyl isothiocyanate, a chemical known to generate ROS [21]. Additionally, De Flora et al. provided a comprehensive review on the mechanisms and protective effects of NAC against DNA damage-related endpoints [22]. Building upon this prior work, the present study aimed to evaluate the overall impact of oxidative stress on β-Dam-induced MN formation. Specifically, we sought to assess if NAC co-treatment could mitigate the genotoxic potential of β-Dam in inducing MN formation using Chinese hamster lung CHL/IU cells and human bronchial epithelial BEAS-2B cells. Furthermore, we aimed to evaluate oxidative stress- and DNA damage-related reporter activities in mouse embryonic stem (mES) cells using the ToxTracker assay, as well as to examine the cellular ROS and GSH levels and γH2AX foci count in BEAS-2B cells using high-content analysis.

It is generally accepted that oxidative stress induced by an oxidant like β-Dam is associated with the clastogenic MoA, as the majority of generated ROS can directly interact with DNA and lead to base modifications, such as 8-oxo-7,8-dihydro-2′-deoxyguanosine [23, 24]. However, as demonstrated by Marcon et al. [25], oxidative stress-induced mitochondrial dysfunction is emerging as another target MoA that induces chromosome displacement and aneuploidy, ultimately leading to MN formation. Therefore, in this study, we also investigated whether β-Dam could affect the mitochondrial membrane potential. Furthermore, we explored if these toxicological results were influenced by co-treatment with NAC, to determine whether the primary MoA for β-Dam-mediated MN induction is oxidative stress.

## Materials and methods

### Chemicals

Reagents and test chemicals were obtained from Thermo Fisher Scientific (Waltham, MA, USA) unless stated otherwise. *N*-Acetyl-l-cysteine (NAC, CAS no.: 616–91-1), menadione sodium bisulfite (CAS no.: 130–37-0), and potassium bromate (KBrO_3_, CAS no.: 7758–01-2) were obtained from Merck/Sigma-Aldrich (St. Louis, MO, USA). Carbonyl cyanide 4-(trifluoromethoxy)phenylhydrazone (FCCP, CAS no.: 370–86-5) was purchased from Tokyo Chemical Industry (Tokyo, Japan). (*Z*)-β-damascone (β-Dam, CAS no.: 23726–92-3) was acquired from Takasago International Corporation (Tokyo, Japan). Mitomycin C (MMC, CAS no.: 50–07-7) was sourced from FUJIFILM Wako Pure Chemical (Osaka, Japan). NAC was dissolved in distilled water, while all other chemicals were dissolved in dimethyl sulfoxide (DMSO).

### MN assays in CHL cells

The MN assay was performed in Chinese hamster lung cells, specifically CHL/IU cells, as previously described [18]. CHL/IU cells were obtained from the Japanese Cancer Research Resources Bank (Osaka, Japan) and maintained in tissue culture dishes containing Minimum Essential Medium supplemented with 10% heat-inactivated bovine serum, penicillin at 100 U/mL, and streptomycin at 100 μg/mL at 37 °C with 5% CO_2_. Cells were seeded at 1,600 cells/well in 200 μL of culture medium in 96-well plates. After pre-incubation for 24 h, the cells were exposed to serially diluted β-Dam for 27 h. MMC was used as the positive control, while 1% DMSO was used as the solvent control. To assess whether β-Dam-induced oxidative stress contributed to MN formation, co-treatment of 10 mM NAC with β-Dam was performed in the MN assay.

Following the 27-h exposure, the supernatant in each well was aspirated and the cells were stained with 13.3 μg/mL Hoechst 33342 (Dojindo Laboratories; Kumamoto, Japan) and 0.833 μg/mL CellMask Orange. After washing twice with Hank’s balanced salt solution, the cells were fixed with 4% paraformaldehyde (FUJIFILM Wako Pure Chemicals) and mounted in 0.005 mg/mL 1,4-diazabicyclo[2.2.2]octane (Tokyo Chemical Industry), 2% glycerol (FUJIFILM Wako Pure Chemicals), 50 mM Tris–HCl buffer (pH 7.4, Takara Bio; Kyoto, Japan) as an antifading agent. High-content images were captured using the CellInsight™ CX5 at 10 × magnification for cell counting and 20 × magnification for MN observation. Excitation (Ex) and emission (Em) wavelengths were selected for Hoechst 33342 (Ex = 375/397 nm and Em = 415/461 nm) and CellMask Orange (Ex = 548/572 nm and Em = 589/619 nm). Forty-nine fields of view were acquired at predetermined positions covering an entire well and cell numbers were counted using the number of Hoechst 33342-stained nuclei. At least 2,000 cells per concentration were examined for micronucleated cells unless severe cytotoxicity was observed.

Relative population doubling (RPD) was chosen as the cytotoxicity parameter and calculated using the following formula [26, 27]:

$$\text{RPD}=\left(\text{number of population doublings in treated cultures}/\text{number of population doublings in control cultures}\right)\times 100$$,

where population doubling = [log(post-treatment cell number/initial cell number)] / log2.

All experiments were independently repeated three times.

### ToxTracker assay

The ToxTracker assay was performed as previously described [15, 28] and conducted at Toxys (Leiden, the Netherlands) in accordance with Good Cell Culture Practice guidelines [29]. mES cells were cultured in gelatin-coated dishes using KnockOut Dulbecco’s Modified Eagle Medium (Toxys) supplemented with 200 µg/mL of G418 in an incubator at 37 °C with 5% CO_2_.

The assay utilized six independent mES green fluorescent protein (GFP) reporter cell lines, each indicative of different genotoxic pathways, including DNA damage (Bscl2 and Rtkn), oxidative stress (Srxn1 and Blvrb), protein damage (Ddit3), and p53 activation (Btg2). These mES cell lines were seeded into gelatin-coated 96-well plates at a density of 4 × 10^4^ cells/well in 200 µL of mES cell culture medium and incubated for 24 h at 37 °C with 5% CO_2_.

Subsequently, the cells were treated with β-Dam for 24 h. To investigate whether oxidative stress is responsible for the genotoxic ability of β-Dam, the antioxidant NAC (10 mM) was added to the cell culture medium together with β-Dam.

After exposure, GFP reporter induction was measured using a Guava easyCyte 10HT benchtop cytometer (Millipore, Merck; Darmstadt, Germany). The median GFP fluorescence value was used to calculate GFP reporter induction in the β-Dam-treated sample relative to the concurrent solvent control cultures without NAC co-treatment. Cytotoxicity in the ToxTracker assay was assessed using the mean relative cell survival rates of the six different reporter cell lines. Triplicate independent experiments were performed.

A positive ToxTracker assay response was defined as a test sample inducing at least a doubling of GFP expression in any of the reporters when the cytotoxicity was ≤ 75%.

### High-content analysis

Experiments were conducted in triplicate with three well replicates per experiment.

#### Cell culture and chemical exposure

Normal human bronchial epithelial BEAS-2B cells were obtained from American Type Culture Collection (Manassas, VA, USA) and cultured in Dulbecco’s Modified Eagle Medium (DMEM) supplemented with 4 mM Glutamax and 10% fetal bovine serum at 37 °C with 5% CO_2_. Cells suspended in 100 μL of culture medium were seeded into each well of a 96-well plate (AGC TECHNO GLASS; Shizuoka, Japan) at 5 × 10^3^ cells/well and incubated overnight at 37 °C with 5% CO_2_. The following day, the supernatants were removed by decantation and the cells were exposed to culture medium containing serially diluted β-Dam. DMSO (1%) was used as the solvent control, while the specific positive control chemical for each analysis was applied as described below. Cells were exposed for 4 h for the intracellular ROS, intracellular GSH, and mitochondrial membrane potential measurements, while they were exposed for 24 h for the MN measurement and γH2AX focus count.

#### MN measurement

Following the 24-h exposure to test chemicals (DMSO or 10 μM MMC), the supernatants were removed and the cells were washed with phosphate-buffered saline (PBS). After fixing the cells with 4% paraformaldehyde for 30 min, they were permeabilized with PBS containing 0.1% Triton X-100 (Sigma-Aldrich) for an additional 30 min. After washing twice with PBS, the cells were stained with 100 μL/well of PBS containing 1 μg/mL Hoechst 33342 and 2 μg/mL CellMask Green for 30 min.

#### Intracellular ROS measurement

Before exposure to each test chemical, 100 μL/well of DMEM containing 20 μM dihydroethidium was added to each well (final concentration: 10 μM dihydroethidium) and incubated for 30 min at 37 °C with 5% CO_2_. After washing twice with the culture medium, the cells were exposed to test chemicals. Menadione sodium bisulfite at 100 μM was used as the positive control. Following the 4-h exposure, the supernatants were removed and the cells were washed with Dulbecco’s phosphate buffered saline (DPBS). Subsequently, the cells were stained with 100 μL/well of DPBS containing 1 μg/mL Hoechst 33342 and 1 μg/mL CellMask Deep Red at 37 °C for 30 min.

#### Intracellular GSH measurement

After the 4-h exposure to test chemicals (DMSO or 100 μM menadione sodium bisulfite), the supernatants were removed and the cells were washed with culture medium. Following medium removal, the cells were stained with 100 μL/well of DPBS containing 100 μM monochlorobimane, 5 μM SYTO82, and 1 μg/mL CellMask Deep Red at 37 °C for 30 min.

#### γH2AX focus counting

Following the 24-h exposure to test chemicals (DMSO or 10 μM MMC), the cells were fixed and permeabilized as described above for MN measurement. After removing PBS containing Triton X-100 from each well, the cells were blocked with PBS containing 1% bovine serum albumin (Sigma-Aldrich) (PBS/BSA) for 30 min. After removing the supernatants, the cells were stained with 50 μL/well of PBS/BSA containing 1 μg/mL anti-γH2AX (anti-γH2AX, phospho Ser139, mouse IgG [9F3], Abcam; Cambridge, UK) for 90 min. After washing with PBS/BSA for 5 min, the cells were further stained with 50 μL/well of PBS/BSA containing 1 μg/mL AlexaFluor647-labeled anti-mouse IgG goat antibody for 90 min. After washing with PBS, the cells were counterstained with PBS containing 1 μg/mL Hoechst 33342 and 2 μg/mL CellMask Green for 90 min.

#### Mitochondrial membrane potential

After the 4-h exposure to test chemicals (FCCP or DMSO), each well received 50 μL JC-10 fluorescent probe (Abcam) and was incubated for 30 min at 37 °C. The cells were fixed with 4% paraformaldehyde for 15 min and counterstained with 1 μg/mL Hoechst 33342 for 30 min.

#### Image capture and data analysis

After washing twice with DPBS, five images of each well were acquired at 10 × magnification for intracellular ROS and GSH assessments, while 25 images of each well were acquired at 20 × magnification for the other measurements using an Operetta CLS high-content imaging system (PerkinElmer; Waltham, MA, USA) (Ex = 490/515 nm and Em = 570/650 nm for dihydroethidium; Ex = 355/385 nm and Em = 430/500 nm for Hoechst 33342; Ex = 615/645 nm and Em = 655/760 nm for CellMask Deep Red; Ex = 355/385 nm and Em = 470/515 nm for monochlorobimane; Ex = 490/515 nm and Em = 570/650 nm for SYTO82; Ex = 490/525 nm and Em = 540/590 nm for JC-10; Ex = 460/490 nm and Em = 500/550 nm for CellMask Green; Ex = 615/645 nm and Em = 655/760 nm for AlexaFluor647). These images were analyzed using Harmony® 4.9 software (PerkinElmer). For cytotoxicity assessment, the RPD for MN measurement and relative cell number for the other measurements were calculated using the images acquired in each measurement. For other toxicological endpoints, up to approximately 1,000 cells per well (a total of 3,000 cells in three wells) were analyzed at each concentration. Metaphase cells were morphologically distinguished from cells in other cell cycle phases and excluded from γH2AX analysis, as metaphase cells are known to express γH2AX independently of the DNA damage response. Fluorescence intensity data obtained from the analysis are presented using arbitrary units, which were normalized relative to the mean of the solvent control group without NAC co-treatment and expressed as a fold change, irrespective of the chemical treatment group.

### Statistical analysis

Prior to conducting the comparative potency analysis, the data underwent screening for cytotoxicity and dose–response patterns. Cytotoxicity was assessed to exclude the reporter response values deemed unreliable, specifically GFP response values for concentrations exhibiting cytotoxicity > 75% in ToxTracker assays, %MN values for concentrations with RPD < 40% in in vitro MN assays, and other measurements for concentrations with the relative cell number < 50%. The results are presented as the mean and standard error of the mean (SEM) of three independent experiments.

For the groups exposed to serially diluted test chemical solutions, Dunnett’s test was employed to assess a significant increase of %MN frequency in both CHL/IU and BEAS-2B cells, intracellular ROS and GSH levels, γH2AX focus counts, and mitochondrial membrane potential. For the groups exposed to the test chemicals at a single concentration, Welch’s *t*-test was used to evaluate a significant increase compared with the solvent control groups. *P*-values < 0.05 were considered statistically significant. Data analysis was conducted using JMP version 10.0.2 (SAS Institute Japan; Tokyo, Japan).

## Results

### NAC completely prevented MN induction in CHL/IU and BEAS-2B cells exposed to β-Dam

**Fig. 2:**
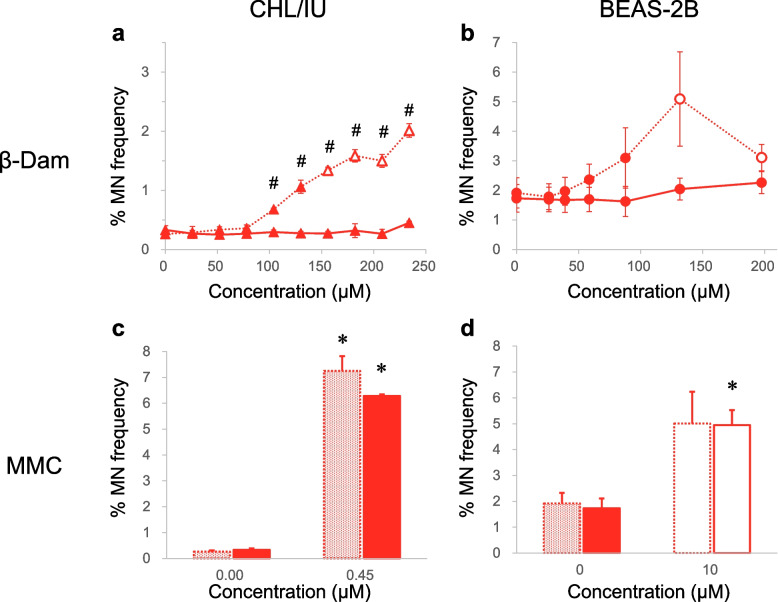
Effect of *N*-acetyl-l-cysteine (NAC) on micronucleus (MN) induction in CHL/IU and BEAS-2B cells exposed to β-damascone (β-Dam) or mitomycin C (MMC). The frequency of micronucleated cells (% MN frequency) was measured after 27 h of exposure to β-Dam or MMC in CHL/IU cells (**a**, **c**). The same measurement was also conducted in BEAS-2B cells (**b**, **d**) after 24 h of exposure to each chemical. Solid and dotted lines represent the values obtained with or without NAC co-treatment, respectively. Closed and open symbols or bars represent the values obtained at concentrations causing more than and less than 40% relative population doubling, respectively. Each result represents the mean and standard error of the mean for three independent experiments. #Significantly different from the solvent control group (*P* < 0.05, Dunnett's test). * Significantly different from the solvent control group (*P* < 0.05, Welch's *t*-test)

Initially, we investigated if oxidative stress is responsible for the misleading positive results in the β-Dam in vitro MN assay by examining the impact of NAC on MN formation in CHL/IU cells, which are validated according to the corresponding Organisation for Economic Co-operation and Development (OECD) test guidelines [30]. We also examined this in BEAS-2B cells, a human bronchial epithelial cell line. We exposed the cells to various concentrations of β-Dam in the absence or presence of 10 mM NAC. The frequency of micronucleated cells and RPD are shown in Fig. 2 and Supplementary Fig. 1, respectively.

In CHL/IU cells, a 27-h exposure to β-Dam alone produced a statistically significant increase in MN frequency at concentration of 104.0 μM or higher. However, these MN increases were completely suppressed by co-treatment with 10 mM NAC (Fig. 2a). A similar suppressive effect was observed in the β-Dam-mediated cytotoxicity (Supplementary Fig. 1a). In BEAS-2B cells exposed to β-Dam for 24 h, concentrations showing RPD values that decreased to less than 40% of those in the solvent control group (Supplementary Fig. 1b) showed a slight, but not statistically significant, increase in MN formation (Fig. 2b). However, with NAC co-treatment, the frequency of micronucleated cells at the highest exposure concentration was comparable to that of the solvent control (Fig. 2b).

In contrast, NAC co-treatment had no evident suppressive effect on MN induction by MMC, a DNA crosslink agent, in both CHL/IU and BEAS-2B cells (Fig. 2c/d), confirming that the protective effect of NAC is limited to the oxidative stress-mediated MN induction in these cells.

These results indicate that oxidative stress is the primary genotoxic MoA of β-Dam, leading to MN formation in mammalian cells.

### NAC suppressed oxidative stress-mediated GFP activation in mES cells exposed to β-Dam in ToxTracker assays

**Fig. 3:**
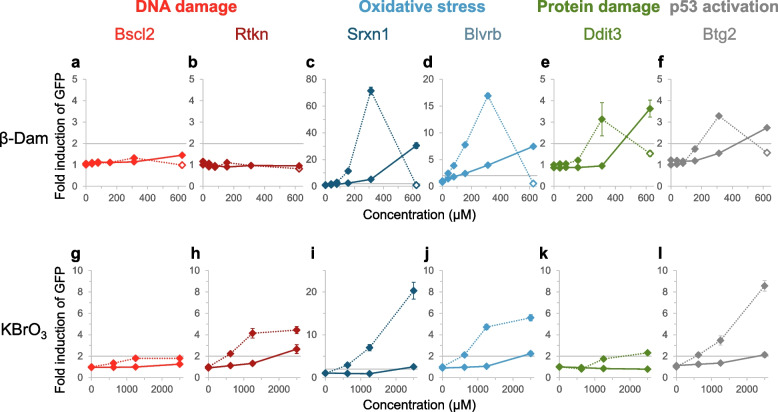
Effect of *N*-acetyl-l-cysteine (NAC) on green fluorescent protein (GFP) activation in mouse embryonic stem reporter cell lines exposed to β-damascone (β-Dam) or potassium bromate (KBrO_3_). The GFP reporter activity fold change was calculated from the mean solvent control values for the six reporter cell lines related to DNA damage [(**a**, **g**) Bscl2 and (**b**, **h**) Rtkn], oxidative stress [(**c**, **i**) Srxn1 and (**d**, **j**) Blvrb], protein damage [(**e**, **k**) Ddit3], and p53 activation [(**f**, **l**) Btg2] after 24 h of exposure to β-Dam or KBrO_3_. Each grey solid line for reporter activation indicates the GFP induction threshold (a factor of two). The colored solid and dotted lines represent the values obtained with or without NAC co-treatment, respectively. The closed and open symbols represent the values obtained at concentrations causing more than and less than 25% relative cell survival, respectively. Each result represents the mean and standard error of the mean for three independent experiments

To further examine the role of oxidative stress in the underlying mechanism of β-Dam-induced in vitro MN formation in mammalian cells, we examined the suppressive effect of NAC using ToxTracker assays. This assay allows for the analysis of oxidative stress, as well as other toxicological MoAs, such as DNA damage, protein damage, and p53 activation, following exposure to various β-Dam concentrations with and without 10 mM NAC. Additionally, six different mES cell lines were treated with KBrO_3_, a known inducer of oxidative stress [31], to confirm the suppressive effect of NAC in this assessment. The GFP fluorescence fold change in each reporter cell line and relative cell survival rates (mean cell number for six different reporter cell lines) after the 24-h exposure to each chemical are shown in Fig. 3 and Supplementary Fig. 2, respectively.

Among the oxidative stress-related reporters, namely the Srxn1-GFP and Blvrb-GFP cell lines, reporter activation increased in a concentration-dependent manner upon exposure to β-Dam, reaching approximately 70- and 17-fold increases, respectively, compared with the solvent control group (Fig. 3c/d). The concentration eliciting the maximum response of the oxidative stress-related GFP reporters (312.5 μM) resulted in 25.9% cell viability (Supplementary Fig. 2a), which did not reach a severe cytotoxic concentration in this assay. However, the highest exposure concentration of 625 µM, which led to 6.6% cell viability, showed a notable decrease in GFP activation (Fig. 3c/d and Supplementary Fig. 2a). Co-treatment with 10 mM NAC clearly suppressed these significant increases in GFP activity in both Srxn1- and Blvrb-expressing reporter cell lines, resulting in only about 5- and 4-fold respective increases at 312.5 μM and about 30- and 7-fold respective increases at 625 μM (Fig. 3c/d). At these concentrations in the presence of NAC, cell survival did not reach a severe cytotoxicity level (Supplementary Fig. 2a). Similarly, concentration-dependent increases in protein damage- and p53 activation-related GFP reporter cell lines (Ddit3-GFP and Btg2-GFP cells) were observed up to 312.5 μM, both of which were suppressed by co-treatment with 10 mM NAC to less than 2-fold compared with the solvent control group (Fig. 3e/f). In contrast, GFP activation by β-Dam exposure was not observed in the DNA damage-related reporter cell lines (Bscl2-GFP and Rtkn-GFP cells) regardless of co-treatment with NAC, even at a severe cytotoxic concentration of 625 μM (Fig. 3a/b and Supplementary Fig. 2a).

Similarly, KBrO_3_ exposure significantly increased GFP activity in a concentration-dependent manner in all reporter cell lines, except for Bscl2-GFP cells (Fig. 3g–l). Even with NAC co-treatment, GFP activation reached significant levels (approximately 2.6-, 2.5-, 2.2-, and 2.1-fold increases over the solvent control) in Rtkn-, Srxn1-, Blvrb-, and Btg2-GFP cell lines at 2500 μM, where cytotoxicity was not observed (about 70% of cell survival compared with the solvent control) (Fig. 3h–j/l and Supplementary Fig. 2b).

These results suggest that oxidative stress plays a central role in various β-Dam-induced toxicological responses. However, unlike KBrO_3_, direct oxidative stress-mediated DNA damage does not appear to be involved in the β-Dam genotoxic MoA.

### NAC inhibited ROS levels and GSH depletion in BEAS-2B cells exposed to β-Dam

**Fig. 4:**
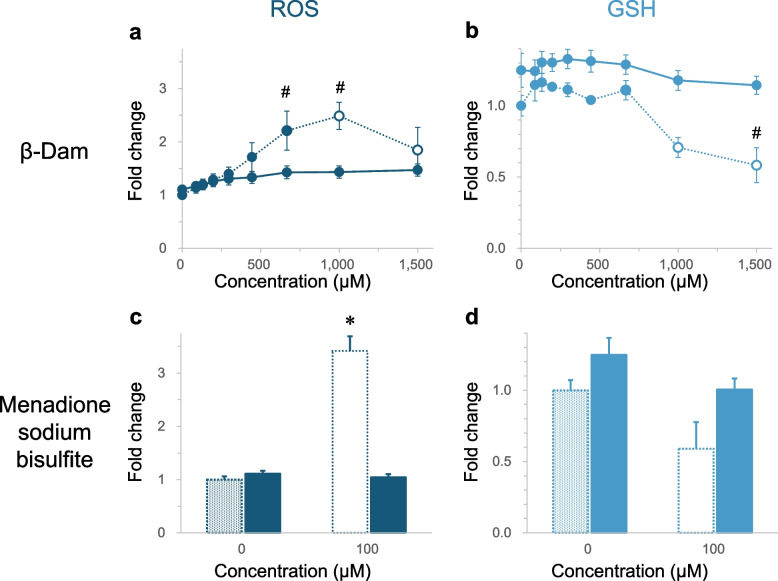
Effect of *N*-acetyl-l-cysteine (NAC) on reactive oxygen species (ROS) and glutathione (GSH) levels in BEAS-2B cells exposed to β-damascone (β-Dam) or menadione sodium bisulfite. The fold changes for ROS (**a**) and GSH (**b**) levels after 4 h of exposure to β-Dam or menadione sodium bisulfite were calculated from the solvent control value without NAC co-treatment. The solid and dotted lines represent the values obtained with or without NAC co-treatment, respectively. The closed and open symbols or bars represent the values obtained at concentrations causing more than and less than 50% relative cell number, respectively. Each result represents the mean and standard error of the mean for three independent experiments. #Significantly different from the solvent control group (*P* < 0.05, Dunnett's test). *Significantly different from the solvent control group (*P* < 0.05, Welch's *t*-test)

Although the ToxTracker assays provided insights into the potential contribution of β-Dam-induced oxidative stress to various toxicological responses, the GFP reporter assay mainly examines changes in gene expression associated with toxicity, such as oxidative stress and DNA damage. Therefore, we subsequently assessed ROS generation using dihydroethidium to measure cytosolic superoxide production [32] and GSH depletion by measuring reduced GSH consumption [33] as more direct evidence of oxidative stress in BEAS-2B cells exposed to β-Dam. BEAS-2B cells were also treated with 100 μM menadione sodium bisulfite, a known superoxide anion generator [34], to confirm the suppressive effect of NAC on ROS generation and GSH depletion. The fold changes of these assessments relative to the solvent control and relative cell counts after a 4-h exposure to each chemical are depicted in Fig. 4 and Supplementary Fig. 3, respectively.

BEAS-2B cells exposure to β-Dam resulted in a statistically significant and concentration-dependent increase in intracellular ROS levels, with the maximum response being approximately 2.5-fold higher than the solvent control detected at 1000 μM, where a certain level of cytotoxicity (43.1% over solvent control in cell counts) was found (Fig. 4a and Supplementary Fig. 3a). Additionally, a concentration-dependent decrease in GSH levels was observed in BEAS-2B cells exposed to β-Dam, with a statistically significant reduction detected at the highest concentration of 1500 μM, where relative the cell count compared with the solvent control was 13.5% (Fig. 4b and Supplementary Fig. 3b). Co-treatment with 10 mM NAC almost completely suppressed this β-Dam-induced ROS increase and GSH decrease (Fig. 4a/b).

A cytotoxic menadione sodium bisulfite concentration of 100 μM (32.2% in cell count, Supplementary Fig. 3c) caused a statistically significant increase in ROS production, which was completely suppressed by NAC co-treatment (Fig. 4c). This demonstrates that NAC co-treatment was able to scavenge the superoxide produced by menadione sodium bisulfite. In contrast to ROS production, the observed GSH decrease following menadione sodium bisulfite exposure did not reach a statistically significant level (66.7% over the solvent control, *P* = 0.315) (Fig. 4d), even at the 100 μM cytotoxic concentration (15.4% in cell count, Supplementary Fig. 3d).

These results suggest that β-Dam can induce cytosolic superoxide production and intracellular GSH depletion to scavenge the superoxide anion, providing more clear evidence that oxidative stress is central to the β-Dam toxicological MoA.

### β-Dam induced MN formation in BEAS-2B cells despite the absence of γH2AX foci

**Fig. 5:**
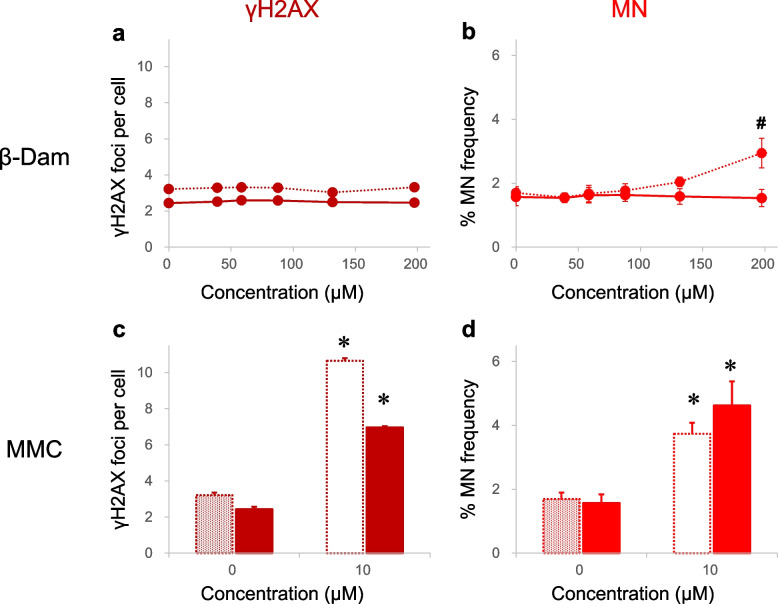
Effect of *N*-acetyl-l-cysteine (NAC) on direct DNA damage and micronucleus (MN) induction in BEAS-2B cells exposed to β-damascone (β-Dam) or mitomycin C (MMC). The fold changes for γH2AX foci per cell as a direct DNA damage marker (**a**) and frequency of micronucleated cells (% MN frequency) (**b**) after 24 h of exposure to β-Dam or MMC were calculated from the solvent control value without NAC co-treatment. The solid and dotted lines represent the values obtained with or without NAC co-treatment, respectively. The closed and open symbols or bars represent the values obtained at concentrations causing more than and less than 50% relative cell number, respectively. Each result represents the mean and standard error of the mean for three independent experiments. #Significantly different from the solvent control group (*P* < 0.05, Dunnett's test). *Significantly different from the solvent control group (*P* < 0.05, Welch's *t*-test)

Although β-Dam induced MN formation through oxidative stress, as demonstrated in Fig. 2, DNA damage-related GFP reporter activation was not detected after a 24-h exposure to β-Dam (Fig. 3). These results raise concerns about the sensitivity of the ToxTracker assay in detecting DNA damage caused by β-Dam-induced oxidative stress. To address this concern, we employed a high-content analysis approach to count the number of γH2AX foci found in the main nuclear region of BEAS-2B cells exposed to β-Dam, as reported by Takeiri et al. [35]. This approach was chosen because γH2AX foci are surrogate markers for a wide range of DNA damage. Previous findings have demonstrated that γH2AX foci are induced not only by DNA double-strand breaks, such as from ionizing radiation and DNA crosslinkers, but also by DNA single-strand breaks after exposure to UV irradiation, topoisomerase inhibitors, and ROS [36–38]. The fold changes of the number of intracellular γH2AX foci relative to the solvent control and frequency of micronucleated cells in BEAS-2B cells after 24 h of exposure to each chemical are depicted in Fig. 5. The relative cell count after chemical exposure is also shown in Supplementary Fig. 4.

High-content analysis revealed that β-Dam exposure, even in the absence of NAC co-treatment, produced similar γH2AX foci counts as the concurrent solvent control (Fig. 5a). This finding did not align with the observation that significant MN induction occurred at the highest exposure concentration of 197.0 μM (Fig. 5b), with almost no cytotoxicity detected (74.1% in cell count, Supplementary Fig. 4a).

However, treatment with the intra- and inter-strand DNA cross-linking genotoxicant MMC displayed a substantial increase of γH2AX foci, with little effect from NAC co-treatment (Fig. 5c), which correlated with the significant MN induction (Fig. 5d).

These results were consistent with the ToxTracker assay data and collectively indicate the absence of DNA damaging potential of β-Dam under the experimental conditions investigated.

### The mitochondrial membrane potential was reduced in BEAS-2B cells exposed to β-Dam

**Fig. 6:**
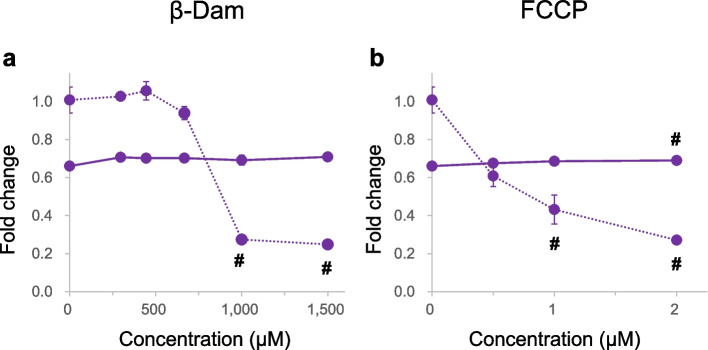
Effect of *N*-acetyl-l-cysteine (NAC) on mitochondrial membrane potential in BEAS-2B cells exposed to β-damascone (β-Dam) or mitochondrial poison. The mitochondrial membrane potential fold changes were expressed as the average fluorescence ratio of polarized and depolarized after 4 h of exposure to β-Dam (**a**) or a mitochondrial poison, carbonyl cyanide 4-(trifluoromethoxy) phenylhydrazone (FCCP) (**b**). These values were calculated from the solvent control value without NAC co-treatment. The solid and dotted lines represent the values obtained with or without NAC co-treatment, respectively. Each result represents the mean and standard error of the mean for three independent experiments. #Significantly different from the solvent control group (*P* < 0.05, Dunnett's test)

The absence of detectable DNA damage indicates that the β-Dam MoA is not related to causing breaks in the DNA strands (clastogenicity), but rather to disrupting the proper chromosome distribution during cell division (aneugenicity). Recent findings by Marcon et al. proposed that mitochondria could serve as an additional target for inducing aneuploidy [25]. They demonstrated that a mitochondrial toxin, carbonyl cyanide 3-chlorophenyl hydrazone, led to chromosome loss through an indirect genotoxic mechanism. This highlights how mitochondrial dysfunction could compromise energy production and consequently disrupt proper mitosis and meiosis progression, processes that require high-energy substrates (ATP) [39]. Considering this hypothesis, we aimed to investigate if β-Dam exposure could trigger mitochondrial dysfunction in BEAS-2B cells by measuring the mitochondrial membrane potential. The fold changes of JC-10 relative to the solvent control and relative cell counts after 4 h of exposure to each chemical are depicted in Fig. 6 and Supplementary Fig. 5, respectively.

Our findings revealed a steep and statistically significant reduction in mitochondrial membrane potential in BEAS-2B cells exposed to β-Dam at almost non-cytotoxic concentrations (1000 and 1500 μM) during the 4-h period (Fig. 6a and Supplementary Fig. 5a). Moreover, this reduction was effectively suppressed when co-treated with 10 mM NAC (Fig. 6a), which was consistent with our prior observations.

To validate our measurement approach employing the JC-10 fluorescent probe, we chose FCCP, a recognized mitochondrial toxin that disrupts the mitochondrial membrane potential [40]. As expected, FCCP induced a dose-dependent reduction in mitochondrial membrane potential after the 4-h exposure, a response that was totally counteracted by co-treatment with NAC (Fig. 6b).

From these data obtained from the mitochondrial membrane potential measurements described above, we conclude that β-Dam can induce mitochondrial dysfunction in BEAS-2B cells. This implies that β-Dam may exert its aneugenic effect, potentially disrupting the precise chromosome distribution pattern during cell division, by affecting mitochondrial function.

## Discussion

In the present study, we aimed to elucidate the MoA underlying the observed MN induction by chemical exposure in vitro. We investigated if it is an indirect effect mediated by oxidative stress by assessing changes in the chemical effects following co-treatment with the antioxidant NAC. Specifically, we focused on β-Dam, known for generating ROS through α, β-unsaturated carbonyl structures, which notably had a potential misleading positive result, as reported in the literature by EFSA.

Following on from the EFSA report indicating that β-Dam tested positive in the in vitro MN assay, we aimed to validate its MN-inducing capacity and the inhibitory effect of NAC using CHL/IU cells, a cell type recommended in the corresponding OECD test guidelines for MN testing. Our findings demonstrated a concentration-dependent increase in micronucleated cells with β-Dam exposure (Fig. 2a). Notably, this increase was significant across a concentration range where considerable cytotoxicity, with a RPD of less than 40%, was observed (Fig. 2a and Supplementary Fig. 1a). This pronounced increase in β-Dam-induced MN formation was completely suppressed by simultaneous co-treatment with 10 mM NAC (Fig. 2a). In addition, MN measurement was also conducted using BEAS-2B cells, a human bronchial epithelial cell line, as β-Dam is commonly used as a fragrance in cosmetics and the respiratory tract is a potential target organ. Even at concentrations inducing maximal MN formation, the increase did not reach statistical significance, possibly indicating that normal p53 levels in BEAS-2B cells and excessive (geno)toxicity hinder further MN induction by arresting cell division. Indeed, ToxTracker assays revealed that p53 activation-related reporter activity was induced by β-Dam exposure in mES cells (Fig. 3f), which may indicate that p53 activation may also occur in BEAS-2B cells upon β-Dam exposure. Furthermore, in BEAS-2B cells, where excessive cytotoxic concentrations did not significantly increase MN (Fig. 2b), co-exposure with NAC and β-Dam completely suppressed the concentration-dependent cytotoxicity, as evidenced by the reduction in RPD (Supplementary Fig. 1b). Unlike β-Dam, MN induction by the DNA cross-linking agent MMC remained unaffected by NAC co-treatment in both CHL/IU and BEAS-2B cells (Fig. 2c/d). Our previous findings with allyl isothiocyanate suggested that oxidative stress-mediated MN induction was suppressed with NAC [18]. Consistent with this, the complete suppression of MN induction by β-Dam with NAC co-treatment in our current study suggested that MN induction is a secondary change mediated by oxidative stress from ROS generation.

Upon observing the complete suppression of MN induction by β-Dam with NAC co-treatment, as shown in Fig. 2a/b, we inferred that the ROS generated by β-Dam were damaging DNA given the anticipated secondary mechanism of MN induction mediated by oxidative stress. Numerous studies using the ToxTracker assay system have recently highlighted examples of MN induction triggered by oxidative and endoplasmic reticulum stress, alongside mechanisms involving direct DNA damage by chemicals [15, 41–43]. Among these, oxidative stress-related reporter activity was notably suppressed in the presence of antioxidants, such as NAC and GSH, as observed with chemicals including KBrO_3_ and sodium (meta)arsenite. This indicates that the mechanism of MN induction by these chemicals is mediated by oxidative stress [15]. In the current study, we performed the ToxTracker assay for β-Dam in the presence of NAC. Our data suggested that oxidative stress-related reporter activity increased in a concentration-dependent manner with β-Dam exposure, which was significantly attenuated in the presence of NAC (Fig. 3c/d).

Our ToxTracker assays revealed upregulation of genes associated with ROS generation. However, it is crucial to note that this assay type primarily assesses changes in the expression of genes involved in defense against oxidative stress, such as Srxn1 and Blvrb [15], rather than directly quantifying specific ROS or antioxidant molecules or enzymes. In our study, we selected dihydroethidium, a fluorescent dye known for its sensitivity in measuring superoxide [32], a precursor to various ROS species, as an indicator of intracellular ROS levels. Additionally, we aimed to assess the reduction in antioxidant molecules by measuring cellular levels of GSH. We observed that β-Dam induced a concentration-dependent increase in ROS levels, even at concentrations where significant cytotoxicity was not observed (relative cell number ≥ 50% of the solvent control) (Fig. 4a and Supplementary Fig. 3a). Moreover, this increase was almost completely suppressed in the presence of NAC (Fig. 4a). The suppressive effect of NAC on ROS elevation was further supported by its ability to prevent ROS generation induced by the superoxide generator, menadione sodium bisulfite (Fig. 4c). For GSH levels, a significant reduction was observed only at the highest tested β-Dam concentration (1500 μM), where the relative cell number dropped to 13.5% (Fig. 4b and Supplementary Fig. 3b). Importantly, this reduction in GSH levels at the highest β-Dam concentration was not observed in the presence of NAC, which suggests that β-Dam-induced oxidative stress is mitigated with sufficient antioxidant activity. The lack of sensitivity of GSH reduction to ROS generation was further supported by the results obtained with the positive control, menadione sodium bisulfite (Fig. 4d), suggesting the involvement of cellular mechanisms in regenerating GSH even with oxidation conditions.

From previous results, oxidative stress could reasonably cause DNA strand breaks. When these breaks exceed the cell's repair capacity, chromosome structural aberrations can occur [23]. Indeed, the DNA damage-related reporter activity, namely Rtkn, increased in response to KBrO_3_-induced oxidative stress (Fig. 3h). KBrO_3_ was included in the ToxTracker assay experiments because it can reportedly induce DNA damage concurrently with oxidative stress, as demonstrated by Ballmaier and Epe [44]. In contrast to KBrO_3_, β-Dam failed to increase DNA damage-related reporter activity (Fig. 3a/b). From the oxidative stress measurements, it was deemed necessary to assess not only changes in the expression levels of relevant genes analyzed by ToxTracker, but also DNA damage itself. Thus, we examined changes in γH2AX, a marker of double-stranded DNA damage. The results showed that β-Dam did not significantly increase γH2AX foci, even at 197.5 μM, where a significant increase in MN formation was observed without severe cytotoxicity (Fig. 5a/b, Supplementary Fig. 4a). This finding contrasts with those using MMC, a DNA crosslinker, which showed a significant increase in γH2AX foci in CHL/IU and BEAS-2B cells, which was almost unaffected by NAC co-treatment in both cell lines (Fig. 5c/d). Our results are consistent with those reported from EFSA, including Ames and in vivo Comet assay negative results [14], strongly suggesting that β-Dam-induced MN was not caused via a clastogenic MoA.

For mechanism classification regarding the induction of micronuclei, our results suggest that β-Dam-mediated MN induction does not occur through a clastogenic mechanism where the compound directly damages DNA. Therefore, it was deemed necessary to investigate it from an aneugenicity perspective. Although the effects on cell division and chromosome distribution have been less studied in relation to oxidative stress, a recent study by Marcon et al. has reported that mitochondrial dysfunction can induce chromosome distribution abnormalities that result in MN induction via an aneugenic mechanism. In their study, primary dermal neonatal human fibroblasts exposed to the mitochondrial toxin cyanide 3-chlorophenyl hydrazone showed no alterations in anaphase shape or multipolar spindle induction, nor any improper activation of the mitotic checkpoint. Instead, chromosome displacement and CREST-positive micronuclei were observed [25]. Considering these findings from Marcon et al., we measured changes in mitochondrial membrane potential in BEAS-2B cells exposed to β-Dam to confirm whether MN induction by β-Dam occurs through a similar aneugenic MoA. A significant and concentration-dependent decrease in mitochondrial membrane potential was observed in cells exposed to β-Dam, similar to cells exposed to carbonyl cyanide 4-(trifluoromethoxy) phenylhydrazone, a known mitochondrial disruptor (Fig. 6b). These outcomes imply that β-Dam can induce micronuclei through an aneugenic effect, not through a clastogenic effect, with mitochondrial dysfunction as the underlying mechanism. Various mechanisms that induce aneuploidy have been extensively studied [45, 46]. These mechanisms include the following: (1) improper regulation of tubulin polymerization and depolymerization, (2) damage to the kinetochore and this impairs correct spindle attachment, and (3) abnormal regulation of the cell cycle. These factors are critical in the induction of aneuploidy. Investigation on these mechanisms will provide important insights into the pathways through which β-Dam exposure induces aneuploidy.

Regarding the mechanisms underlying mitochondrial dysfunction, β-Dam is hypothesized to directly disrupt mitochondrial membranes. The α, β-unsaturated carbonyl moiety of β-Dam reacts with the thiol group of glutathione and with the cysteine residues in certain proteins [47], potentially leading to the disruption of mitochondrial membrane structures. However, within the scope of our investigation, we did not find any studies suggesting that β-Dam directly and physically damages the mitochondrial membrane through such mechanisms. Furthermore, if β-Dam has a destructive effect on other lipid bilayer membranes in addition to the mitochondrial membrane, it could also damage the plasma membrane. Nevertheless, in our high-content analysis of MN formation and ROS production, no cellular images suggested plasma membrane disruption following β-Dam exposure (data not shown). The disturbance of mitochondrial function by β-Dam exposure needs to be further clarified from mechanistic and confirmative perspectives. Future studies focusing on epigenetic modifications in mitochondrial DNA and changes in intracellular calcium concentrations are anticipated to provide a more comprehensive and precise understanding of the mechanisms underlying mitochondrial dysfunction [48], in addition to changes in the mitochondrial membrane potential (Fig. 6).

This aneugenic MoA from mitochondrial dysfunction is newly discovered and recently reported [25], so only very few case reports have described similar mechanisms of action. However, some flavor chemicals have shown a significant increase in oxidative stress reporter activity in ToxTracker assays without increasing DNA damage-related reporter activity like β-Dam. Thakkar et al. [49] reported that certain flavor chemicals can significantly increase oxidative stress-related reporter activity, but not that of DNA damage-related reporters. Of the four chemicals they examined, at least 2-octen-4-one and *p*-methoxycinnamaldehyde showed a significant dose-dependent increase in oxidative stress-related reporter activity of both Srxn1 and Blvrb. However, neither Bscl2 nor Rtkn DNA damage-related reporter activity increased more than twofold up to the highest concentration. Interestingly, *p*-methoxycinnamaldehyde has been reported to induce oxidative stress, but not DNA damage, and to inhibit mitochondrial function [50], resulting in MN formation through aneugenic and clastogenic pathways [51]. Furthermore, another known mitochondrial inhibitor, rotenone, induces MN by an aneuploidy-based mechanism associated with the inhibition of mitochondrial function [52]. Taking into consideration these two chemicals in addition to a mitochondrial poison, carbonyl cyanide 3-chlorophenyl hydrazone [25], the mechanism of the aneugenic mode by β-Dam may share commonalities with other well-studied genotoxicants. Future research should focus on comparative studies with these chemicals and further investigate the common genotoxic mode of action.

## Conclusions

In the present study, we found that β-Dam exposure could induce ROS production in mammalian cells, which then led to MN formation through a non-DNA-reactive MoA (aneugenicity). Moreover, the antioxidant agent, NAC, significantly suppressed ROS levels and the subsequent toxicological events induced by β-Dam. These findings suggest that oxidative stress may play a role in an aneugenic MoA via mitochondria dysfunction, leading to MN formation in mammalian cells. This mechanism is distinct from the generation of oxidative DNA damage, which is theoretically associated with a clastogenic MoA involving direct DNA interactions.

## Supplementary Information

Supplementary Material 1.

## Data Availability

The data generated and analyzed during the current study are available from the corresponding author on reasonable request.

## Abbreviations

β-Dam: β-Damascone
DMSO: Dimethyl sulfoxide
DPBS: Dulbecco’s phosphate buffered saline
EFSA: European Food Safety Authority
Em: Emission
Ex: Excitation
FCCP: Carbonyl cyanide 4-(trifluoromethoxy) phenyl-hydrazone
GFP: Green fluorescent protein
GSH: Glutathione
KBrO_3_: Potassium bromate
mES: Mouse embryonic stem
MN: Micronucleus
MMC: Mitomycin C
MoA: Mode of action
NAC: *N*-Acetyl-L-cysteine
OECD: Organisation for Economic Co-operation and Development
PBS: Phosphate-buffered saline
PBS/BSA: PBS containing 1% bovine serum albumin
ROS: Reactive oxygen species
RPD: Relative population doubling
