# Supplementary material for: Exploring the role of oxidative stress and mitochondrial dysfunction in β-damascone-induced aneuploidy
Source: Genes Environ. 2024 Nov 25;46:25. doi: 10.1186/s41021-024-00319-3 (PMC11590541; doi:10.1186/s41021-024-00319-3)
Supplement: Supplementary file 1 — Supplementary Material 1. [file 41021_2024_319_MOESM1_ESM.docx]

**Supplementary Information**


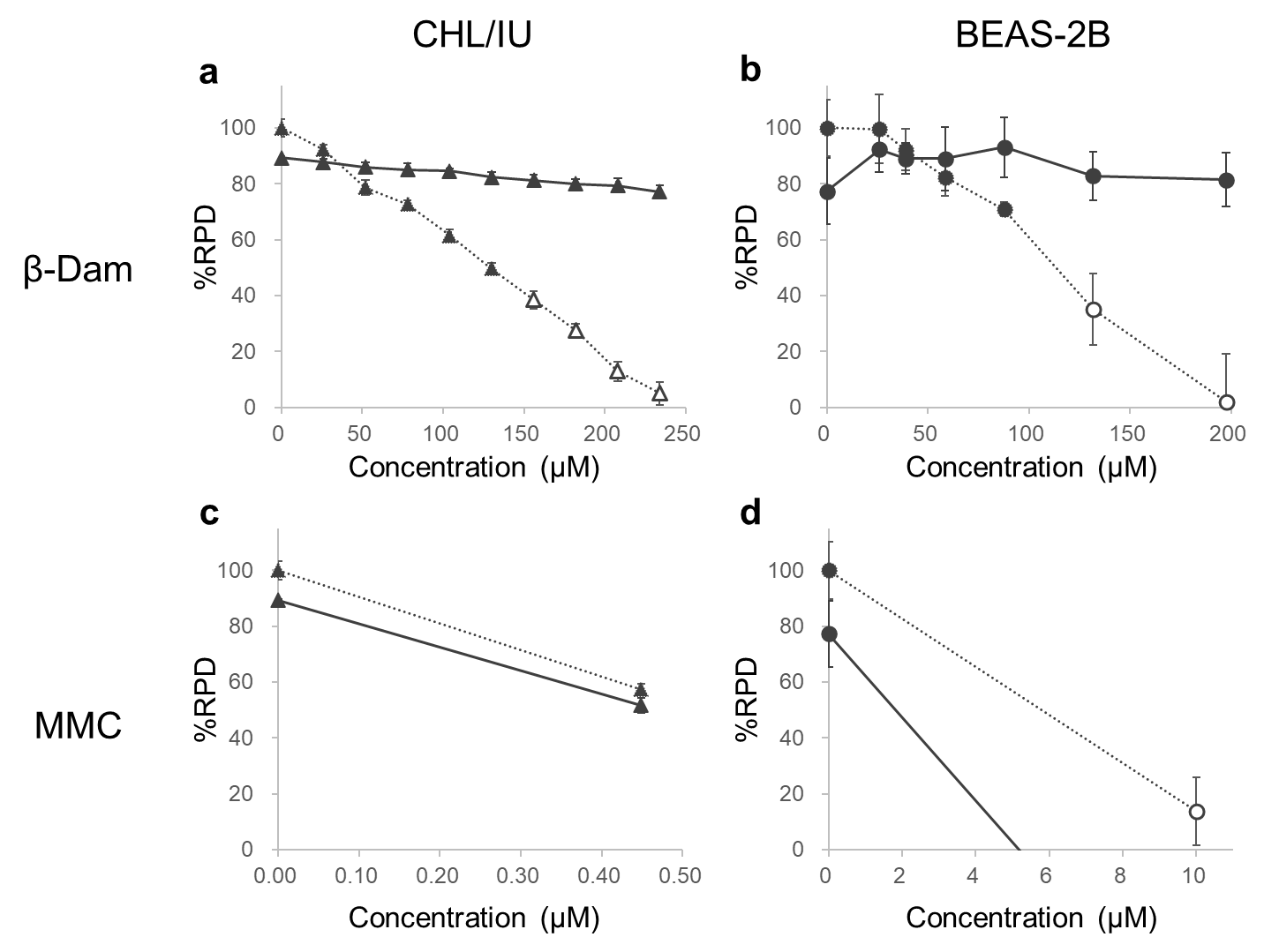


**Supplementary Figure 1. Effect of *N*-acetyl-l-cysteine (NAC) on relative population doubling (RPD) in CHL/IU and BEAS-2B cells exposed to β-damascone (β-Dam) or mitomycin C (MMC).**

RPD values (%RPD) were measured after 27 hours of exposure to β-Dam or MMC in CHL/IU cells (**a**, **c**). The same measurements were conducted in BEAS-2B cells (**b**, **d**) after 24 hours of exposure to each chemical. The solid and dotted lines represent the values obtained with or without NAC co-treatment, respectively. The closed and open symbols represent the values obtained at concentrations causing more than and less than 40% RPD, respectively. Each result represents the mean and standard error of the mean for three independent experiments.

**
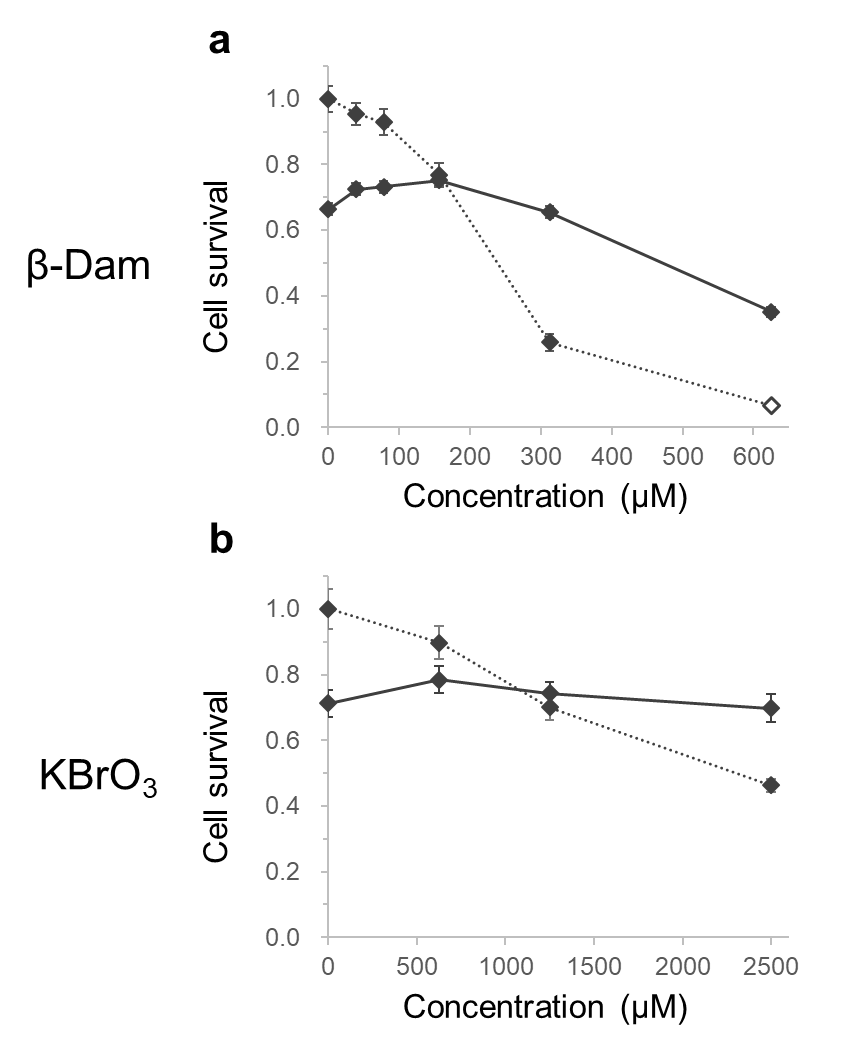
**

**Supplementary Figure 2. Effect of *N*-acetyl-l-cysteine (NAC) on relative cell survival in mouse embryonic stem reporter cell lines exposed to β-damascone (β-Dam) or potassium bromate (KBrO_3_).**

Cell survival levels after 24 hours of exposure to β-Dam (**a**) or KBrO_3_ (**b**) were calculated from the normalized cell count. The solid and dotted lines represent the values obtained in the co-treatment with or without NAC, respectively. The closed and open symbols represent the values obtained at concentrations causing more than and less than 25% relative cell survival, respectively. Each result represents the mean and standard error of the mean for three independent experiments.


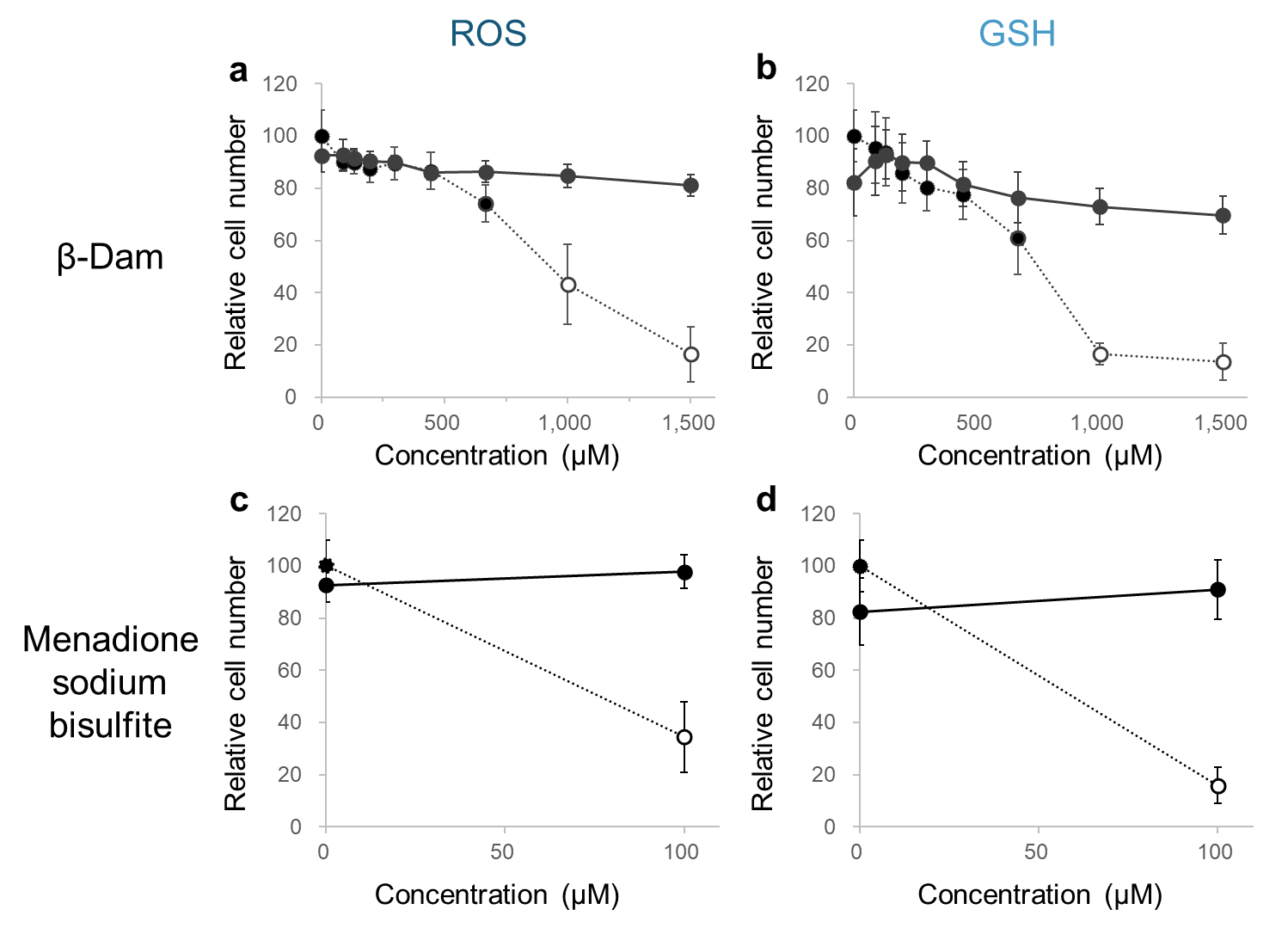


**Supplementary Figure 3. Effect of *N*-acetyl-l-cysteine (NAC) on the relative cell number in BEAS-2B cells exposed to β-damascone (β-Dam) or menadione.**

The relative cell number was calculated after 24 hours of exposure to β-Dam or menadione sodium bisulfite in BEAS-2B cells for measuring reactive oxygen species (ROS) (**a**, **c**) and glutathione (GSH) (**b**, **d**) levels. The solid and dotted lines represent the values obtained with or without NAC co-treatment, respectively. The closed and open symbols represent the values obtained at concentrations causing more than and less than 50% relative cell number, respectively. Each result represents the mean and standard error of the mean for three independent experiments.


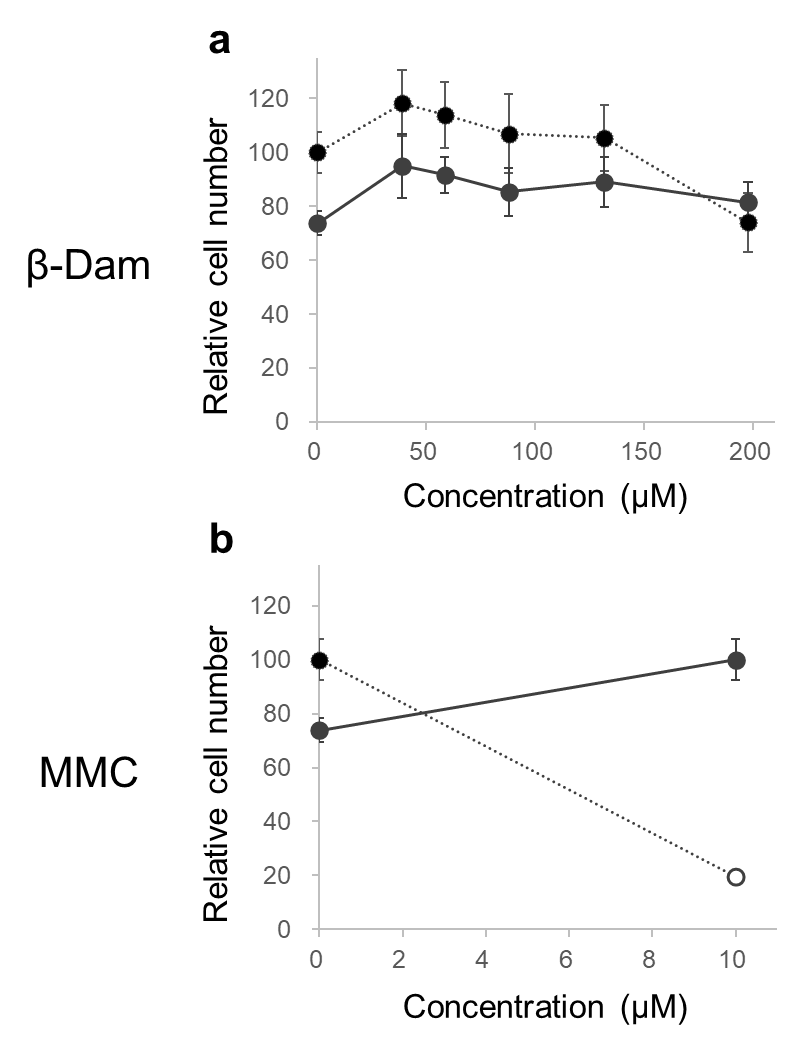


**Supplementary Figure 4. Effect of *N*-acetyl-l-cysteine (NAC) on the relative cell number in BEAS-2B cells exposed to β-damascone (β-Dam) or mitomycin C (MMC).**

The relative cell number was calculated after 24 hours of exposure to β-Dam (**a**) or MMC (**b**) in BEAS-2B cells for measuring γH2AX foci. The solid and dotted lines represent the values obtained with or without NAC co-treatment, respectively. The closed and open symbols represent the values obtained at concentrations causing more than and less than 50% relative cell number, respectively. Each result represents the mean and standard error of the mean for three independent experiments.


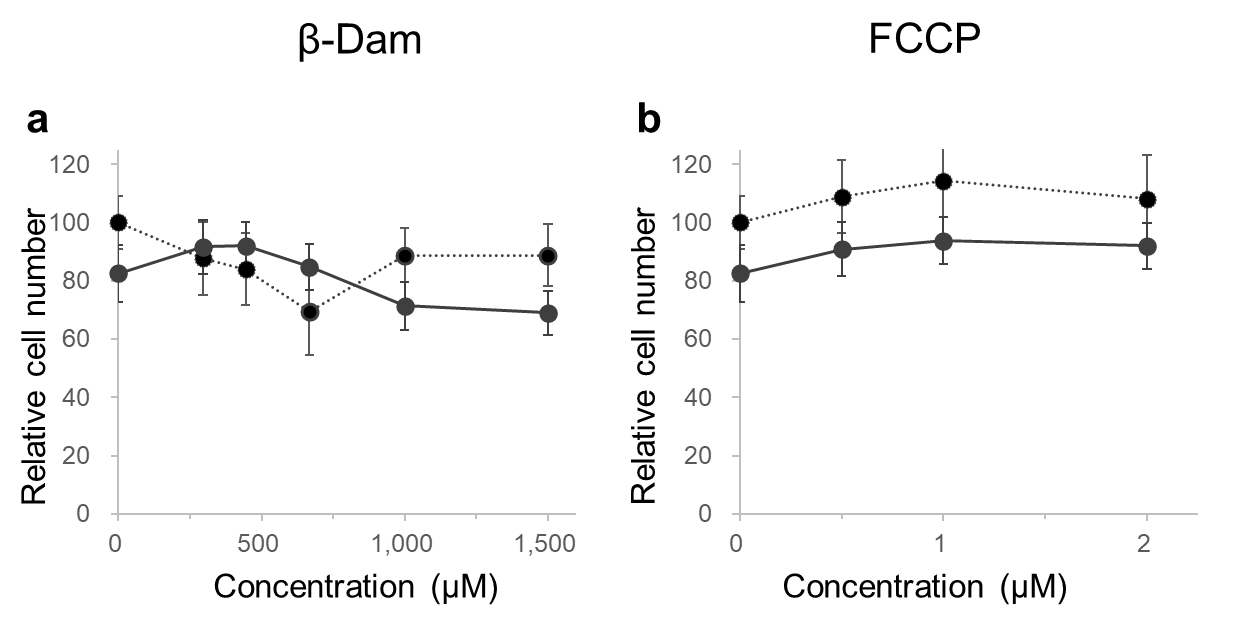


**Supplementary Figure 5. Effect of *N*-acetyl-L-cysteine (NAC) on the relative cell number in BEAS-2B cells exposed to β-damascone (β-Dam) or mitochondrial poison.**

The relative cell number was calculated after 4 hours of exposure to β-Dam (**a**) or a mitochondrial poison, carbonyl cyanide 4-(trifluoromethoxy) phenyl-hydrazone (FCCP) (**b**) in BEAS-2B cells. The solid and dotted lines represent the values obtained with or without NAC co-treatment, respectively. Each result represents the mean and standard error of the mean for three independent experiments.
